# Straight to the point: evaluation of a Point of Care Information (POCI) resource in answering disease-related questions

**DOI:** 10.5195/jmla.2024.1770

**Published:** 2024-01-16

**Authors:** Rachel L. Wasserman, Diane L. Seger, Mary G. Amato, Zoe Co, Aqsa Mugal, Angela Rui, Pamela M. Garabedian, Marlika Marceau, Ania Syrowatka, Lynn A. Volk, David W. Bates

**Affiliations:** 1 rwasserman@bwh.haravrd.edu, Division of General Internal Medicine, Brigham and Women's Hospital, Boston, MA and Massachusetts College of Pharmacy and Health Sciences (MCPHS), Boston, MA; 2 dseger@mgb.org, Clinical and Quality Analysis, Mass General Brigham, Somerville, MA; 3 mamato1@bwh.harvard.edu, Division of General Internal Medicine, Brigham and Women's Hospital, Boston, MA; 4 zco@bwh.harvard.edu, Division of General Internal Medicine, Brigham and Women's Hospital, Boston, MA; 5 amugal@mgb.org, Clinical and Quality Analysis, Mass General Brigham, Somerville, MA; 6 arui@mgb.org, Division of General Internal Medicine, Brigham and Women's Hospital, Boston, MA; 7 pmgarabedian@partners.org, Clinical and Quality Analysis, Mass General Brigham, Somerville, MA; 8 mmarceau@mgb.org, Clinical and Quality Analysis, Mass General Brigham, Somerville, MA; 9 asyrowatka@bwh.harvard.edu, Division of General Internal Medicine, Brigham and Women's Hospital, Boston, MA and Harvard Medical School, Boston, MA; 10 lavolk@partners.org, Clinical and Quality Analysis, Mass General Brigham, Somerville, MA; 11 dbates@bwh.harvard.edu, Division of General Internal Medicine, Brigham and Women's Hospital, Boston, MA, Clinical and Quality Analysis, Mass General Brigham, Somerville, MA and Harvard Medical School, Boston, MA

**Keywords:** Evidence-based information, Clinical Decision Support Systems, point of care resources, Information Retrieval

## Abstract

**Objective:**

To evaluate the ability of DynaMedex, an evidence-based drug and disease Point of Care Information (POCI) resource, in answering clinical queries using keyword searches.

**Methods:**

Real-world disease-related questions compiled from clinicians at an academic medical center, DynaMedex search query data, and medical board review resources were categorized into five clinical categories (complications & prognosis, diagnosis & clinical presentation, epidemiology, prevention & screening/monitoring, and treatment) and six specialties (cardiology, endocrinology, hematology-oncology, infectious disease, internal medicine, and neurology). A total of 265 disease-related questions were evaluated by pharmacist reviewers based on if an answer was found (yes, no), whether the answer was relevant (yes, no), difficulty in finding the answer (easy, not easy), cited best evidence available (yes, no), clinical practice guidelines included (yes, no), and level of detail provided (detailed, limited details).

**Results:**

An answer was found for 259/265 questions (98%). Both reviewers found an answer for 241 questions (91%), neither found the answer for 6 questions (2%), and only one reviewer found an answer for 18 questions (7%). Both reviewers found a relevant answer 97% of the time when an answer was found. Of all relevant answers found, 68% were easy to find, 97% cited best quality of evidence available, 72% included clinical guidelines, and 95% were detailed. Recommendations for areas of resource improvement were identified.

**Conclusions:**

The resource enabled reviewers to answer most questions easily with the best quality of evidence available, providing detailed answers and clinical guidelines, with a high level of replication of results across users.

## INTRODUCTION

Access to evidence-based drug and disease information is essential for health care professionals to optimize patient care [1]. Electronic information resources, accompanying conventional practices of textbook use and colleague consults, have become a standard approach used to guide clinical care decisions [2–3]. The clinical decision support system of Point-of-Care Information (POCI) resources supports health care providers in answering clinical questions in a timely manner with curated evidenced-based information [4–6].

Although several published research studies are available regarding satisfaction when using clinical information resources, few studies have sufficiently evaluated the ability of POCI resources to answer real-world clinical questions [7–10]. For example, in Nickum et al, three POCI resources of Nursing Reference Center Plus, ClinicalKey for Nursing, and UpToDate were evaluated by nursing staff to answer three clinical questions and then rate their experience based on currency, relevancy, layout, navigation, labeling, and use of filters [10]. In Bradley-Ridout et al, medical residents each answered four clinical questions and compared the accuracy, time to answer, user confidence, and user satisfaction between two POCI resources of UpToDate and DynaMed [8]. However, these studies were limited either by the small number of questions searched or use of questions from medical board review study guides or textbooks rather than questions asked in a direct patient care setting.

DynaMed and Micromedex with Watson, also known as DynaMedex (Merative and EBSCO), is an evidence-based drug and disease information resource intended to help inform clinical decisions at the point of care (POC) [11–13]. DynaMed is a peer-reviewed clinical content resource with information on disease topics, health conditions, abnormal findings, disease evaluation, differential diagnosis, and disease management [11]. Micromedex is a comprehensive medication information resource with detailed drug monographs, information on drug-drug interactions, and management of drug reactions [12]. The merging of DynaMed and Micromedex into a combined tool, DynaMedex, brought drug and disease information into a single resource to help health care providers in making informed clinical decisions [11–13]. We previously evaluated the application's ability to answer clinical questions in 11 categories (adverse drug reaction/toxicity, alternative medicine, disease, therapeutics and pharmacology, dosing/pharmacokinetics, drug administration, interactions, monitoring/laboratory tests, pregnancy/lactation/breastfeeding, product availability and drug identification, stability/compatibility) and nine specialties (cardiology, critical care, endocrinology, hematology-oncology, infectious disease, neurology, internal medicine, pharmacy, and nursing) [14]. DynaMedex was found to be a useful resource in answering questions in that study, however, the questions were mostly focused on drug therapy, with only a limited number of disease-related clinical questions [14]. The objective of this study was to evaluate the ability of the DynaMedex POCI resource to answer real-world disease-related clinical queries using keyword searches.

## METHODS

The study team reviewers included three research pharmacists with a background in clinical pharmacy and informatics. Two pharmacists had prior experience using DynaMedex. The other pharmacist had experience using DynaMed and Micromedex as separate information resources. The study was conducted from May 2022 to April 2023 and the study team was provided access to DynaMedex during that timeframe. This research project was reviewed and approved by the Mass General Brigham institutional review board (2022P002066).

### Developing and Searching Clinical Disease-related Questions

The study team compiled a list of 265 real-world disease-related questions using multiple resources. Some questions were submitted by or compiled during interviews with specialty clinicians at our academic medical center to identify questions that occurred in their practices. Other questions were created by the research pharmacists using DynaMedex's data of search terms which were anonymized to the research team, and other questions were based on content from medical board review resources [15–17]. All compiled questions were reviewed by physician specialists to confirm clinical relevance and accuracy. The questions were categorized into five clinical categories (complications & prognosis, diagnosis & clinical presentation, epidemiology, prevention & screening/monitoring, and treatment) and targeted six specialties (cardiology, endocrinology, hematology-oncology, infectious disease, internal medicine, and neurology). The number of questions based on clinical category and specialty are summarized in Table 1.

**Table 1:** Overall count of question categories by specialty

Targeted Specialty	Total Questions n (%)	Complications & Prognosis n (%)	Diagnosis n (%)	Epidemiology n (%)	Prevention & Screening/Monitoring n (%)	Treatment n (%)
**Total Questions**	265	38	61	48	50	68
**Cardiology**	42 (16)	7 (18)	8 (13)	7 (15)	10 (20)	10 (15)
**Endocrinology**	34 (13)	6 (16)	7 (11)	6 (13)	6 (12)	9 (13)
**Hematology-Oncology**	34 (13)	6 (16)	6 (10)	5 (10)	7 (14)	10 (15)
**Infectious Disease**	47 (18)	6 (16)	9 (15)	12 (25)	6 (12)	14 (21)
**Internal Medicine**	62 (23)	6 (16)	18 (30)	8 (17)	14 (28)	16 (24)
**Neurology**	46 (17)	7 (18)	13 (21)	10 (21)	7 (14)	9 (13)

The real-world disease-related questions were randomly divided among the three pharmacists for review. Each question was independently reviewed by two of the three between December 2022 to February 2023. Reviewers searched for answers to the questions by entering free text into the search field of the POCI resource and then selected the most appropriate monograph from the options that were returned.

### Data Collection Categories

**Figure 1:**
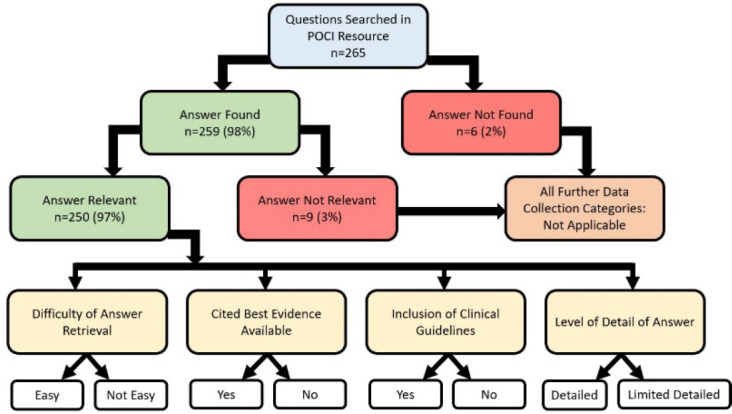
Study methods

*Note: Comments of reviewers were also recorded for every question.

After conducting a literature review of studies evaluating drug information resources, the following categories were created to evaluate the availability, relevance, difficulty of answer retrieval, quality of answers, whether clinical guidelines were included, and level of detail of answers provided [18–22]. Search results were independently evaluated by pharmacists based on if the answer was found (yes, no), whether the answer was relevant to the question (yes, no), difficulty in finding an answer (easy, not easy), cited best evidence available (yes, no), clinical practice guidelines included (yes, no), and level of detail of the evidence provided (detailed, limited details). Study methods are summarized in Figure 1.

1. *Availability of answer*: Each pharmacist documented which search terms were used and the monograph where the answer was found for each question. The pharmacist evaluated if an answer was found or not. If both reviewers did not find an answer, then the further data collection categories were considered not applicable (n/a).
2. *Relevance of the information found*: The pharmacists evaluated relevance of answers if found. If the answer was relevant (yes), the answer fully addressed the question, or if the answer was not relevant (no), some parts of the question were unanswered or did not fully answer the question. If both reviewers found an answer was not relevant, then the further data collection categories were considered not applicable (n/a).
3. *Difficulty of answer retrieval*: The difficulty of answer retrieval was categorized as easy or not easy. Those rated not easy required more than a few minutes of searching, entering multiple search terms, and/or looking at multiple monographs to find the answer.
4. *Cited best evidence available*: Best available evidence in general was considered to be randomized clinical trials or systematic reviews. However, for certain situations where it may not have been possible due to ethical concerns (e.g., pregnancy, lactation), observational studies or case reports may have been considered best available evidence.
5. *Inclusion of clinical guidelines*: Each reviewer assessed if clinical guidelines were available or not available.
6. *Level of detail of answer*: The level of detail of an answer was identified based on if the answer was detailed or not detailed. Detailed answers provided a large amount of information to answer the question such as details on research done to support the answer. Limited detailed answers provided scant information to answer the question such as a single sentence.
7. *Reviewer's comments*: Overall insights and recommendations for improvement were captured about each question searched.

Once all questions were searched, the data was consolidated and analyzed among the three reviewer pharmacists. Differences assessing whether an answer was found were adjudicated through discussion among all three pharmacists until an agreement was reached. Descriptive statistics were calculated to summarize the data.

## RESULTS

### All Questions Regardless of Specialty or Category

The overall results for all questions regardless of specialty or category are summarized in Table 2. An answer was found for 259 of the total 265 questions (98%) in DynaMedex. Both reviewers found the answer for 241 questions (91%), both did not find the answer for 6 questions (2%), and 18 questions were found by one reviewer but not the second reviewer (7%). The difference in finding an answer between reviewers was due to the search terms used in the POCI resource. Select question and answer examples from the data collection categories are summarized in Table 3. Both reviewers found a relevant answer 97% of the time when an answer was found. Of the 250 relevant answers found, 68% were easy to find, 97% cited best quality of evidence available, 72% provided clinical guidelines, and 95% were detailed.

**Table 2:** Overall count of questions regardless of specialty or category

If an answer was found (n=265)	n (%)
Yes	241 (91)
No	6 (2)
Yes and No = where one reviewer found the answer, but the other reviewer did not	18 (7)
**Relevance of the information for answers found (n=259)**	
Yes	250 (97)
No	9 (3)
**Difficulty in finding a relevant answer (n=250)**	
Easy	170 (68)
Not Easy	22 (9)
Mixed = where one reviewer rated the answer as easy, but the other reviewer rated the answer as not easy	58 (23)
**Cited best available evidence for relevant answers found (n=250)**	
Yes	243 (97)
No	7 (3)
**Inclusion of clinical practice guidelines for relevant answers found (n=250)**	
Yes	180 (72)
No	70 (28)
**Level of detail provided for relevant answers found (n=250)**	
Detailed	237 (95)
Limited Details	13 (5)

**Table 3:** Select examples from the data collection categories

**Availability of answer**		
**Example:**	**Question:**	**Explanation:**
A question where neither reviewer found an answer	“After receiving COVID-19 vaccine how long should patients wait before having a mammogram for preventive screening for breast cancer?”	The Society of Breast Imaging released updated guidelines in February 2022 with a new recommendation of no delay between vaccine and a screening mammograph, which was not found in DynaMedex.
A question where one reviewer found an answer, but the second reviewer did not	“What is the number of patients needed to treat to see a benefit of spironolactone in Heart Failure for Reduced Ejection Fraction?”	One reviewer did not find an answer when searching “spironolactone for heart failure,” but the other reviewer found the answer using the search term “aldosterone antagonists for heart failure,” where a summary of a randomized trial with spironolactone was listed in the monograph. Difference in finding an answer between reviewers was due to reviewer's search terms.
**Relevance of the information found**		
**Example:**	**Question:**	**Explanation:**
A question where the answer was rated as not relevant	“When should a Coronary Artery Calcium (CAC) assessment in an intermediate risk individual be repeated if the initial score is zero?”	The Dynamedex monograph cited The American College of Cardiology/American Heart Association 2019 guidelines on primary prevention of cardiovascular disease and cited an answer of 5–10 years. However, this answer differed from the cardiology specialist answer of 3–5 years. Additional searching through other resources found that the answer of 3–5 years matched another POCI resource citing a Multiethnic Study of Atherosclerosis.
**Difficulty of answer retrieval**		
**Example:**	**Question:**	**Explanation:**
A question that was rated as not easy by both reviewers	“All of the following viruses can cause latent infections EXCEPT,” and answer choices listed various infections such as Hepatitis A, B, or C.	This took a considerable amount of time and effort as both reviewers needed to search each of the individual choices to find a virus that did not cause for latent infections.
A question where the answer was rated as not easy to find for one reviewer and easy for the other	“A patient has upper quadrant abdominal pain, chills, vomiting and confusion. An abdomen ultrasound was done showing multiple stones in the gallbladder. Which is the most likely diagnosis?” Answers included acute cholecystitis, hepatitis, liver abscess, etc.	One reviewer had to look up each answer choice in the monographs and read through clinical and diagnostic findings for each, many of which overlapped between the conditions. However, this question was easy for the second reviewer using the search terms “stones in the gallbladder” from the question and that directed the reviewer to the Choledocholithiasis monograph which answered the question. Therefore, the rating for the question was different due to the reviewer's search terms used in DynaMedex.
**Cited best evidence available**		
**Example:**	**Question:**	**Explanation:**
A question with an answer that cited best evidence available	“Which of the following is the most common cardiac complication in children born to mothers with Systemic Lupus Erythematosus?”	DynaMedex lists observational studies and case reports for level of evidence, which is the highest that can be achieved in pregnancy outcomes.
A question with an answer that did not cite the best evidence available	“A patient is started on Riluzole for Amyotrophic Lateral Sclerosis. How often and what labs should be monitored while on this medication?”	The answer cited only the manufacturer's package insert. In this case, the product information is not the best available evidence. Best evidence available would have been inclusion of randomized trials used for the drug to be approved or systematic review articles.
**Inclusion of clinical guidelines**		
**Example:**	**Question:**	**Explanation:**
A question with an answer that included clinical guidelines	“Which is the most appropriate first test to confirm the diagnosis of patients with diabetes mellitus?” Answer choices included random plasma glucose level and Hemoglobin A1c.”	DynaMedex listed guideline recommendations from the American Diabetes Association (ADA) for diagnostic criteria of diabetes mellitus.
**Level of detail of answer**		
**Example:**	**Question:**	**Explanation:**
A question with an answer that was detailed	“What is the most validated test to screen and monitor for severity of cognitive impairment in patients with dementia?”	DynaMedex listed comparisons of validated cognitive screening tests for dementia including the Mini-Mental State Exam (MMSE) which is the most widely used cognitive screening test in primary care. Numerous studies were listed alongside various guidelines (EFNS-ENS, Canadian Task Force on preventative health care, and NINCDS-ADRDA practical guidelines).
A question with an answer that was not detailed	“A mutation in which of the following genes is responsible for CADASIL (Cerebral autosomal dominant arteriopathy with subcortical infarcts and leukoencephalopathy) disorder?”	DynaMedex provided an answer of a sequence alternation in NOTCH3 gene. While it may have answered the question, this question was rated as not detailed since it was limited in the information provided as no other information about the gene was included.

Reviewer comments were generally positive regarding the application's ability in finding answers. For example, reviewers found the direct website links for disease related monographs to be beneficial. Experience with the product did not affect the ability to find an answer as the number of answers not found were distributed among the searches of the research team. A few recommendations for areas of improvement of the resource were identified: (1) providing direct website links for all studies including within the drug monographs, (2) documenting the date when the monograph was last updated, and (3) enhancing search term recognition when search terms were slightly misspelled or had dashed punctuation as often no search results would appear.

### Results by Clinical Categories

Questions were further analyzed within five clinical categories (complications & prognosis, diagnosis & clinical presentation, epidemiology, prevention & screening/monitoring, and treatment). The results by clinical category are summarized in Table 4. An answer was found by both reviewers in treatment (97%; n=66), complications & prognosis (92%; n=35), prevention & screening/monitoring (92%; n=46), epidemiology (88%; n=48), and diagnosis & clinical presentation (85%; n=52).

**Table 4:** Questions results by clinical category when a relevant answer was found

Clinical Categories	Total Questions n (%)	Relevant answers found n (%)	Easy to find answer n (%)	Not Easy to find answer n (%)	Mixed where one reviewer found the answer easily and the other did not n (%)	Cited Best Available Evidence n (%)	Did Not Cite Best Available Evidence n (%)	Guidelines Available n (%)	Guidelines Not Available n (%)	Detailed n (%)	Limited Details n (%)
**Complications & Prognosis**	38 (14)	35 (92)	24 (69)	1 (3)	10 (29)	34 (97)	1 (3)	25 (71)	10 (29)	34 (97)	1 (3)
**Diagnosis & Clinical Presentation**	61 (23)	58 (95)	31 (53)	13 (22)	14 (24)	58 (100)	0	41 (71)	17 (29)	52 (90)	6 (10)
**Epidemiology**	48 (18)	43 (90)	26 (60)	6 (14)	11 (26)	43 (100)	0	20 (47)	23 (53)	39 (91)	4 (9)
**Prevention & Screening/Monitoring**	50 (19)	46 (92)	36 (78)	0	10 (22)	44 (96)	2 (4)	39 (85)	7 (15)	44 (96)	2 (4)
**Treatment**	68 (26)	68 (100)	53 (78)	2 (3)	13 (19)	64 (94)	4 (6)	55 (81)	13 (19)	68 (100)	0

For treatment category questions, an answer was found for all 68 questions (100%) by at least one reviewer, and these were all considered relevant answers by the reviewers. For 2 of the 68 questions, an answer was found by one reviewer but not the second reviewer (3%). Difference in finding an answer between reviewers was due to the search terms used including misspellings. For example, one reviewer found the answer with the correctly spelled search term, “lomentospora proliFICANS,” but the other reviewer did not find an answer with the misspelled search term “lomentospora proliFERICAN.” When search terms were misspelled, DynaMedex did not show any results. Additional examples are summarized in Table 3.

When one reviewer found an answer easily and the other reviewer did not find the answer easily, this was recorded as mixed in Table 4. Difference in finding an answer between reviewers was due to the reviewer's search terms. An answer was more likely to be classified as easily found and detailed in the prevention & screening/monitoring, treatment, and complications & prognosis categories. In contrast, those not easily found or with mixed level of difficulty between users were more likely to be in the diagnosis & clinical presentation and epidemiology categories. When an answer had the best quality of evidence available, the answer tended to also provide clinical guidelines, such as in the complications & prognosis and diagnosis & clinical presentation categories. For epidemiology, all questions were answered by the best quality of evidence available, often observational studies, and clinical guidelines were also available for about half of the epidemiological questions.

### Results by Specialty Area

Questions were also analyzed in each of the six targeted specialties (cardiology, endocrinology, hematologyoncology, infectious disease, internal medicine, and neurology). The results by specialty are summarized in Table 5. An answer was found by both reviewers in infectious disease (89%; n=42), cardiology (90%; n=38), endocrinology (91%; n=31), hematology-oncology (91%; n=31), neurology (91%; n=42) and internal medicine (92%; n=57).

**Table 5:** Questions results by specialty area when a relevant answer was found

Targeted Specialty	Total Questions n (%)	Relevant answers found n (%)	Easy to find answer n (%)	Not Easy to find answer n (%)	Mixed where one reviewer found the answer easily and the other did not n (%)	Cited Best Available Evidence n (%)	Did Not Cite Best Available Evidence n (%)	Guidelines Available n (%)	Guidelines Not Available n (%)	Detailed n (%)	Limited Details n (%)
**Cardiology**	42 (16)	38 (90)	23 (61)	2 (5)	13 (34)	37 (97)	1 (3)	36 (95)	2 (5)	38 (100)	0
**Endocrinology**	34 (13)	33 (97)	25 (76)	1 (3)	7 (21)	31 (94)	2 (6)	30 (91)	3 (9)	30 (91)	3 (9)
**HematologyOncology**	34 (13)	32 (94)	21 (66)	5 (16)	6 (19)	32 (100)	0	25 (78)	7 (22)	32 (100)	0
**Infectious Disease**	47 (18)	45 (96)	31 (69)	4 (9)	10 (22)	44 (98)	1 (2)	30 (67)	15 (33)	40 (89)	5 (11)
**Internal Medicine**	62 (23)	59 (95)	39 (66)	6 (10)	14 (24)	57 (97)	2 (3)	41 (69)	18 (31)	57 (97)	2 (3)
**Neurology**	46 (17)	43 (93)	31 (72)	4 (9)	8 (19)	42 (98)	1 (2)	18 (42)	25 (58)	40 (93)	3 (7)

The reviewers were able to find the answers in each specialty area easily with a range of 61-76%, with the answers easiest to find in endocrinology. There were mixed levels of difficulty between users to find the answers in cardiology (34%). The cardiology, hematologyoncology, and internal medicine specialties provided detailed answers over 97% of the time, while the infectious disease and endocrinology specialties provided limited detailed answers about 10% of the time. All specialties presented the best quality of evidence available, with hematology-oncology having the best quality of evidence available (100%) and the lowest specialty being endocrinology (94%). Clinical guidelines were widely available in cardiology (95%), endocrinology (91%), hematology-oncology (78%), infectious disease (67%), internal medicine (69%), and not as often available in neurology (42%), which likely reflects available published clinical guidelines within these specialties.

### A Selection of Results by Clinical Category and Specialty Area

We analyzed questions by clinical categories and targeted specialties. An answer was not found by either reviewer for six questions with a breakdown of cardiology epidemiology questions (n=2), neurology complications and prognosis questions (n=2), a hematology-oncology prevention and screening/monitoring question (n=1), and an infectious disease epidemiology question (n=1).

The best quality of evidence was not available mainly in the treatment sections, for example with 22% endocrinology treatment questions (n=2), 10% cardiology treatment (n=1), and 7% infectious disease treatment (n=1). Limited details were given for endocrinology diagnosis & clinical presentation (43%; n=3), infectious disease diagnosis & clinical presentation (38%; n=3), neurology epidemiology (20%; n=2), infectious disease complications & prognosis (17%; n=1), internal medicine epidemiology (14%; n=1), neurology prevention & screening/monitoring, infectious disease epidemiology (9%; n=1), and internal medicine prevention & screening/monitoring (8%; n=1).

## DISCUSSION

We evaluated a commercial POCI application with a focus on real-world disease-related clinical questions and found that it generally performed well, although we also identified opportunities for improvement. Overall, the reviewers were able to answer real-world disease-related queries using keyword searches in the application with ease, and much of the time it provided the best evidence available, included detailed answers, and offered access to clinical guidelines. Such resources are likely to become increasingly important in care delivery going forward.

Previous studies have compared POCI resources to one another or evaluated the satisfaction of using a certain POCI product. For example, Bradley-Ridout et al and Baxter SL et al, evaluated the ability of two POCI resources to answer questions developed using Medical Knowledge Self-Assessment Program (MKSAP), a resource for medical education. They also evaluated ease of finding answers and quality of evidence available [4,8]. Although we used similar resources as a base for developing some questions, we attempted to make our questions more real-world and clinically relevant by consulting clinical specialist physicians. Other studies looked at features included by POCI resources but did not evaluate the ability of the resource to answer questions [2, 23].

### Strengths

This study has several strengths. Overall, there was a high level of consistent agreement among the reviewers for questions regardless of specialty or clinical category, such as in answer found, relevant answer, cited best quality available, and if an answer was detailed. Differences in finding an answer between reviewers were due to the reviewer's search terms used. Over 250 clinically relevant questions were generated from multiple sources including clinical specialists and covered a wide variety of categories and specialties. Results of this study should be of interest to readers of this journal who may be considering this resource in their library collections. The resource provided drug and disease related support in one integrated tool which can be used to support clinical decisions at the point of care. Libraries should consider the information from our study along with comparisons for subscriptions for the service at their institution to costs for comparable products. Finally, the study participants were able to easily find answers supported by high quality evidence to most of their queries.

### Limitations

The study was conducted at a single academic medical center using local staff (consultants and researchers) to develop the questions, so the types of questions included may differ from other health care settings. A small sample of questions were used for each specialty and category, which may not be representative of all queries searched in the POCI resource. There may not have been enough numbers of questions for some specialties to get a representative sample to assess the tool and we did not adjust for the differences in the number of questions by specialty or category in the analysis. The complexity of the questions was not evaluated, which may have affected the availability of the answers. While answers to disease-related questions were found, validation of the application's use in the clinical setting as a POCI reference should be further studied. Although pharmacists were searching the questions for this study, this resource has been used by other health professions for usability testing [9]. Further evaluation should be confirmed in real time direct patient care settings.

We evaluated a commercial POCI application which provided evidence about drugs and diseases and found across a range of categories and specialties it enabled reviewers to answer most disease-related questions easily with the best quality of evidence available, providing detailed answers and clinical guidelines. We also identified opportunities for improvement including recognition of misspelled search terms, documenting the date of monograph updates, and providing direct website links for studies mentioned for all references.

## Data Availability

Data for this study is in the attached appendices. Exact text of the questions used could not be provided due to proprietary nature of the content.

## SUPPLEMENTAL FILES

**Appendix A:** Dynamex Results Deidentified
